# Supplementary material for: Managing migraine with over-the-counter provision of triptans: the perspectives and readiness of Western Australian community pharmacists
Source: PeerJ. 2019 Dec 16;7:e8134. doi: 10.7717/peerj.8134 (PMC6921984; doi:10.7717/peerj.8134)
Supplement: Supplemental Information 2 [file peerj-07-8134-s002.pdf]

	1	2	3	4	5	6	7
1. Age	21-30	31-40	41-50	51-60	61+		
2. Gender	Male	Female	Other/prefer not to say				
3. Years practising	<6	6-20	>20				
4. Role in pharmacy	sole proprietor	partner proprietor	PIC	Manager	employee pharm	other	
5. Size of operation	small	large					
6. Setting	isolated	shopping strip	city centre	medical centre	small shopping centre	large shopping centre	other
7. Location	city	suburb	rural	remote			
8. Location in relation to nearest Drs	co-located	<100m	101-500m	501m-1k	>1k		
9. Postcode							
10. Accredited?	yes	yes (undergoing accred)	no				
11. Migraine sufferer?	yes	no					
12.	strongly agree	agree	don’t know/unsure	disagree	strongly disagree		
13. Current OTC 1st line	paracetamol	aspirin	other NSAIDs	combined para/NSAID	combined para/metoclopramide	refer	other
14. Current OTC preference if 1st line was CI or did not work	paracetamol	aspirin	other NSAIDs	combined para/NSAID	refer	other	
15. 1st line OTC if triptans available	paracetamol	aspirin	other NSAIDs	combined para/NSAID	triptans	other	
16. Ranking	paracetamol	aspirin	other NSAIDs	Triptans	opioids	other	

17. Times per month emergency supply	0	<2	3-4	>5			
18. Meds other than analgesics	metoclopramide	prochlorperazine	other	metoclopramide + prochlorperazine	metoclopramide + other	prochlorperazine + other	metoclopramide + prochlorperazine + other
19. Comp meds	feverfew	ginger	coQ10	peppermint	other		
20-25	strongly agree	agree	don't know/unsure	disagree	strongly disagree		
