# Supplementary material for: Managing migraine with over-the-counter provision of triptans: the perspectives and readiness of Western Australian community pharmacists
Source: PeerJ. 2019 Dec 16;7:e8134. doi: 10.7717/peerj.8134 (PMC6921984; doi:10.7717/peerj.8134)
Supplement: Supplemental Information 3 [file peerj-07-8134-s003.pdf]

School of Pharmacy and Biomedical Sciences

Curtin University

The Readiness of Western Australian Community Pharmacists to Manage Migraine Including Over-The-Counter Provision of Triptans

This questionnaire should take about 15 minutes to complete. Your responses will be treated with strict confidentiality. Please, if possible, answer all questions**. Please respond to the following questions by ticking the relevant box, circling the relevant number, or writing on the line, where appropriate.**

Section A: Demographics

1. What is your age?
☐ 21–30 ☐ 31–40 ☐ 41–50 ☐ 51–60 ☐ 61+

2. What is your gender?
☐ Male ☐ Female ☐ Other/prefer not to say

3. How many years have you been practising as a pharmacist in Australia?
☐ <6 ☐ 6–20 ☐ >20

4. Which of the following best describes your role in this pharmacy?
☐ Sole proprietor ☐ Partner proprietor ☐ Pharmacist in charge ☐ Manager ☐ Employee pharmacist
☐ Other (please specify) ___________________

5. Which of the following best describes the size of operation of this pharmacy?
☐ Small (turnover ≤$2m per annum) ☐ Large (turnover >$2m per annum)

6. Which of the following best describes the setting of this pharmacy?
☐ Isolated ☐ Shopping strip ☐ City centre ☐ Medical centre
☐ Small shopping center (15-50 shops) ☐ Large shopping center (>50 shops)
☐ Other (please specify) __________________

7. Which of the following best describes the location of this pharmacy?
☐ City ☐ Suburb ☐ Rural ☐ Remote

8. Which of the following best describes the location of this pharmacy in relation to the nearest doctor's surgery or clinic?
☐ Co-located ☐ < 100 metres ☐ 101 – 500 metres ☐ 501 metres – 1 kilometre ☐ > 1 kilometre

9. Please provide the pharmacy location's postcode: ________

10. Are you an accredited pharmacist or are you undergoing accreditation?
☐ Yes, accredited pharmacist ☐ Yes, underdoing accreditation
☐ No, not an accredited pharmacist or undergoing accreditation

11. Do you suffer from migraine?
☐ Yes ☐ No

Section B: Migraine

12. For each of the statements below, circle the response that best characterises **your opinion** about the statement; where:
1 = Strongly Agree, 2 = Agree, 3 = Don't Know/Unsure, 4 = Disagree, and 5 = Strongly Disagree

		Strongly Agree	Agree	Don't Know/ Unsure	Disagree	Strongly Disagree
a.	Migraine is caused by the vasodilation of cranial vessels	1	2	3	4	5
b.	Migraine is caused by a dysfunction of brain stem nuclei	1	2	3	4	5
c.	Migraine is always preceded by an aura	1	2	3	4	5
d.	People with migraine are more likely to experience serious comorbidities (e.g. psychological illness, diabetes, hypertension)	1	2	3	4	5
e.	Pharmacists should refer patients seeking pain relief for undiagnosed migraine	1	2	3	4	5
f.	Pharmacists should refer patients with a headache that differs from their usual migraine/pattern of migraine	1	2	3	4	5
g.	Pharmacists should refer patients who have an aura without headache	1	2	3	4	5
h.	Pharmacists should refer children under 12 years of age with migraine	1	2	3	4	5
i.	Pharmacists should refer patients if their migraine has lasted more than 72 hours (untreated or unsuccessfully treated)	1	2	3	4	5
j.	Pharmacists should refer patients who have had a recent head injury and are requesting treatment for migraine	1	2	3	4	5
k.	There are specific criteria that differentiate headache from migraine	1	2	3	4	5

Section C: Treatment Options

13. Which **ONE** of the following medications/classes of medications would you currently recommend **OTC** as **first line** treatment for an acute migraine episode?
☐ Paracetamol ☐ Aspirin ☐ Other NSAIDs ☐ Combined paracetamol/NSAID
☐ Combined paracetamol/metoclopramide ☐ I would refer the patient to a doctor
☐ Other (please specify) _______

14. Which **ONE** of the following medications/classes of medications would you select if the **first line** treatment that you would usually recommend was contraindicated or did not work?
☐ Paracetamol ☐ Aspirin ☐ Other NSAIDs ☐ Combined paracetamol/NSAID
☐ I would refer the patient to a doctor ☐ Other (please specify) _______

15. Which **ONE** of the following medications/classes of medications would you recommend as **first line** treatment if triptans were OTC, for the treatment of an acute migraine episode?
☐ Paracetamol ☐ Aspirin ☐ Other NSAIDs ☐ Combined paracetamol/NSAID ☐ Triptans
☐ Other (please specify) _______

16. Please rank the following medications/classes of medications according to how commonly patients at your pharmacy request them OTC for the treatment of acute migraine episodes, with 1 being most commonly requested to 6 being least commonly requested.
__ Paracetamol __ Aspirin __ Other NSAIDs __ Triptans __ Opioids
__ Other (please specify) _______

17. How many times **PER MONTH** do you supply a triptan as an emergency supply?
☐ 0 ☐ ≤ 2 ☐ 3 – 4 ☐ ≥ 5

18. What medications, other than analgesics, do you commonly recommend for patients with an acute migraine episode?
☐ Metoclopramide ☐ Prochlorperazine ☐ Other (please specify) _______

19. What complementary or topical products do you commonly recommend for patients with an acute migraine episode?

☐ Feverfew ☐ Ginger ☐ Co-enzymeQ10 ☐ Peppermint

☐ Other (please specify) _______

20. The following statements relate to **triptans**.
For each of the statements below, circle the response that best characterises **your opinion** about the statements; where:
1 = Strongly Agree, 2 = Agree, 3 = Neither Agree nor Disagree, 4 = Disagree, and 5 = Strongly Disagree
Note: the term 'first-time migraine' refers to migraine in a patient who has not previously experienced migraine or migraine undiagnosed by a doctor, and the term 'repeat migraine' refers to migraine that has been previously diagnosed by a doctor.

		Strongly Agree	Agree	Neither Agree nor Disagree	Disagree	Strongly Disagree
a.	Triptans reduce migraine pain	1	2	3	4	5
b.	Triptans alone reduce nausea and vomiting associated with migraine	1	2	3	4	5
c.	Triptans reduce photophobia and phonophobia associated with migraine	1	2	3	4	5
d.	Triptans are most effective if taken at the onset of headache	1	2	3	4	5
e.	If a patient fails to respond to a triptan, there is benefit to trialing a different triptan	1	2	3	4	5
f.	Triptans cannot be used in patients with ischemic heart disease	1	2	3	4	5
g.	Patients can currently readily obtain a doctor's appointment for triptans for a repeat migraine episode	1	2	3	4	5
h.	For an undiagnosed migraine, obtaining a triptan immediately OTC from a pharmacy in a holiday location in Australia (or a pharmacy other than the patient's usual pharmacy), would be the best management option	1	2	3	4	5
i.	For a repeat migraine, obtaining a triptan immediately as an emergency supply from a pharmacy in a holiday location in Australia (or a pharmacy other than the patient's usual pharmacy), where a prescription has not been previously dispensed, would be the best management option	1	2	3	4	5

Section D: Attitudes Towards Down-Scheduling to Schedule 3

21. The following statements are **reasons put forward** by the Australian National Drug and Poisons Scheduling Committee when they deferred or rejected previous proposals to down-schedule a triptan. For each of the statements below, circle the response that best characterises **your opinion** about the statement; where:
1 = Strongly Agree, 2 = Agree, 3 = Don't Know/Unsure, 4 = Disagree, and 5 = Strongly Disagree

		Strongly Agree	Agree	Don't Know/ Unsure	Disagree	Strongly Disagree
a.	Any migraine episode requires a medical diagnosis	1	2	3	4	5
b.	There is no suitable algorithm or questionnaire for pharmacists to use to diagnose migraine	1	2	3	4	5
c.	Triptans need to be used with caution in patients with cardiovascular disease	1	2	3	4	5
d.	Triptans may mask symptoms of more serious conditions such as meningitis	1	2	3	4	5
e.	Drug interactions involving triptans render them unsuitable for down-scheduling to S3	1	2	3	4	5
f.	There is no public health need to down-schedule a triptan due to emergency supply provisions	1	2	3	4	5

22. For each of the statements below in relation to **OTC provision of triptans**, circle the response that best characterises **your opinion** about the statement; where:
1 = Strongly Agree, 2 = Agree, 3 = Neither Agree nor Disagree, 4 = Disagree, and 5 = Strongly Disagree

		Strongly Agree	Agree	Neither Agree nor Disagree	Disagree	Strongly Disagree
a.	Triptans should only be available OTC if a pharmacist is involved in the sale	1	2	3	4	5
b.	If triptans were made available OTC, they should only be available in a maximum pack size of two standard doses	1	2	3	4	5
c.	Triptans are safe to use when provided OTC	1	2	3	4	5
d.	Pharmacists are currently able to supply OTC some medications with serotonergic activity	1	2	3	4	5

23. For each of the statements below in relation to **diagnosis of migraine**, circle the response that best characterises **your opinion** about the statement; where:
1 = Strongly Agree, 2 = Agree, 3 = Neither Agree nor Disagree, 4 = Disagree, and 5 = Strongly Disagree
Note: the term 'first-time migraine' refers to migraine in a patient who has not previously experienced migraine or migraine undiagnosed by a doctor, and the term 'repeat migraine' refers to migraine that has been previously diagnosed by a doctor.

		Strongly Agree	Agree	Neither Agree nor Disagree	Disagree	Strongly Disagree
a.	Most pharmacists can accurately diagnose a first-time migraine	1	2	3	4	5
b.	Most pharmacists can accurately diagnose repeat migraine	1	2	3	4	5
c.	Most doctors can accurately diagnose a first-time migraine	1	2	3	4	5
d.	Most doctors can accurately diagnose repeat migraine	1	2	3	4	5
e.	Most pharmacists can accurately identify when it is appropriate to refer patients with migraine for medical review	1	2	3	4	5
f.	Patients who are migraine sufferers recognize the symptoms of migraine onset	1	2	3	4	5

24. For each of the statements below in relation to **training and resources**, circle the response that best characterises **your opinion** about the statement; where:
1 = Strongly Agree, 2 = Agree, 3 = Neither Agree nor Disagree, 4 = Disagree, and 5 = Strongly Disagree
Note: the term 'first-time migraine' refers to migraine in a patient who has not previously experienced migraine or migraine undiagnosed by a doctor, and the term 'repeat migraine' refers to migraine that has been previously diagnosed by a doctor.

		Strongly Agree	Agree	Neither Agree nor Disagree	Disagree	Strongly Disagree
a.	Pharmacists would require additional training to manage first-time migraine OTC	1	2	3	4	5
b.	Pharmacists would require additional training to manage repeat migraine OTC	1	2	3	4	5
c.	Pharmacists would require additional tools (i.e. questionnaire or algorithm) to diagnose first-time migraine	1	2	3	4	5
d.	Pharmacists would require additional tools (i.e. questionnaire or algorithm) to diagnose repeat migraine	1	2	3	4	5
e.	There are sufficient resources available to pharmacists to support first-time migraine diagnosis	1	2	3	4	5
f.	There are sufficient resources available to pharmacists to support repeat migraine diagnosis	1	2	3	4	5

25. For each of the statements below in relation to the **potential outcomes of down-scheduling of triptans to Schedule 3**, circle the response that best characterises **your opinion** about the statement; where:
1 = Strongly Agree, 2 = Agree, 3 = Neither Agree nor Disagree, 4 = Disagree, and 5 = Strongly Disagree

		Strongly Agree	Agree	Neither Agree nor Disagree	Disagree	Strongly Disagree
a.	Down-scheduling a triptan may improve timely patient access to effective migraine medication	1	2	3	4	5
b.	If a triptan was down-scheduled, pharmacists may be more able to assist people in the treatment of migraine	1	2	3	4	5
c.	Down-scheduling would increase the risk of overuse of triptans	1	2	3	4	5
d.	Triptans are too potent for OTC prescribing	1	2	3	4	5
e.	Down-scheduling a triptan may reduce burden on doctors	1	2	3	4	5
f.	Down-scheduling a triptan may ease the financial burden on patients with migraine	1	2	3	4	5
g.	Down-scheduling a triptan may ease the financial burden on society due to patients with migraine taking less time off work	1	2	3	4	5

Please provide any further comments you have about the **potential outcomes of down-scheduling of triptans to Schedule 3**.

____

____

____

THANK YOU FOR YOUR CONTRIBUTION

Please kindly return the completed questionnaire in the reply-paid envelope enclosed by the 23rd of April 2018.
